# New US device versus imaging US to assess tumor-in-brain

**DOI:** 10.1186/s41016-020-00205-1

**Published:** 2020-08-05

**Authors:** Jacob Halevy-Politch, Menashe Zaaroor, Alon Sinai, Marius Constantinescu

**Affiliations:** 1 Aerospace Eng., Technion City, Haifa, Israel; 2 School of Medicine, Technion I.I.T., Haifa, Israel; 3 Department of Neurosurgery, Rambam HCC, Haifa, Israel

**Keywords:** Neurosurgery, Tumor, Ultrasound, Pulse-echo, Residual thickness

## Abstract

**Background:**

Applying ultrasonic imaging system during surgery requires the poring of saline, performing the measurement, and acquiring data from its display—which requires time and is highly “performer dependent,” i.e., the measure is of a subjective nature. A new ultrasonic device was recently developed that overcomes most of these drawbacks and was successfully applied during tumor-in-brain neurosurgeries. The purpose of this study was to compare the two types of US devices and demonstrate their properties.

**Methods:**

The study was performed in the following stages: (i) an ex vivo experiment, where slices of the muscle and brain of a young porcine were laid one on top the other. Thicknesses and border depths were measured and compared, using the two types of US instruments. (ii) During human clinical neurosurgeries, tumor depth was compared by measuring it with both devices. (iii) Following the success of stages (i) and (ii), using solely the new US device, the tumor thickness was monitored while its resection.

Correlation, Pearson’s coefficient, average, mean, and standard deviation were applied for statistical tests.

**Results:**

A high correlation was obtained for the distances of tissue borders and for their respective thicknesses. Applying these ultrasonic devices during neurosurgeries, tumor depths were monitored with high similarity (87%), which was also obtained by Pearson’s correlation coefficient (0.44). The new US device, thanks to its small footprint, its remote measurement, and the capability of monitoring intraoperatively and in real-time, provides the approach to tumor’s border before its complete resection.

**Conclusions:**

The new US device provides better accuracy than an ultrasonic imaging system; its data is objective; it enables to *control* the residual tumor thickness during its resection, and it is especially useful in restricted areas. These features were found of great help during a tumor-in-brain surgery and especially in the final stages of tumor’s resection.

## Background

Medical ultrasound (US) monitoring systems are known for many years due to their capability to distinguish between different soft tissues in a living human body, including the difference between a healthy and a tumor tissue. This property is based on their ability to distinguish between ultrasonic (US) characteristics of each tissue [such as US *attenuation* (AT) and its *velocity* (Speed-of-Sound = SOS), their US *reflection* (Re) (named also “Pulse echoes” (PE), in case of pulse transmission), and *transmission* (Tr)]. These abilities have an advantage in neurosurgery [1–5], such as in procedures of monitoring and removing a tumor from a healthy tissue. The tumor type glioma, including low-grade glioma (LGG), performs a special challenge in monitoring and resection, as it grows invasively and it is more difficult to separate it from healthy brain tissue. Another neurosurgical requirement is to obtain the monitored data intraoperative (IO) and in real-time (RT), in order to control (with a neurosurgeon-in-the-loop) the surgery and especially the resecting process [5–8]. Neurosurgeons were the first one to apply scanning the brain with an US A-Mode imaging device [9]. However, the A-Mode devices were not developed further, since the US imaging systems replaced them. The available US imaging systems are feasible but are time consuming and provide suboptimal results [1, 2]. It turns out that the dimensions of an US transducer limit its applicability in many neurosurgical cases [10]. This was found as a drawback, especially during a resection process, which is performed in many cases in a restricted and confined area. In these cases, it is essential that the residual tumor thicknesses should be monitored IO and in RT. In conventional US systems, a thin layer of conductive US media like normal saline (NS) [5%, 25 °C] is required, which stops a surgical process; moreover, a thin layer of NS was not found feasible in restricted area applications. It was also recognized that for a proper operation of the US system and its image analysis, specialization is essential [10], and some of these instruments are relatively expensive [1, 2, 5, 10].

Other currently available tools include intraoperative magnetic resonance imaging (MRI) and computer tomographic imaging (CTI or CT), comprised of more complicated to operate and expensive equipment in the operating room (OR). They also have limitations in their ability for an accurate decision-making process [10].

**Fig. 1:**
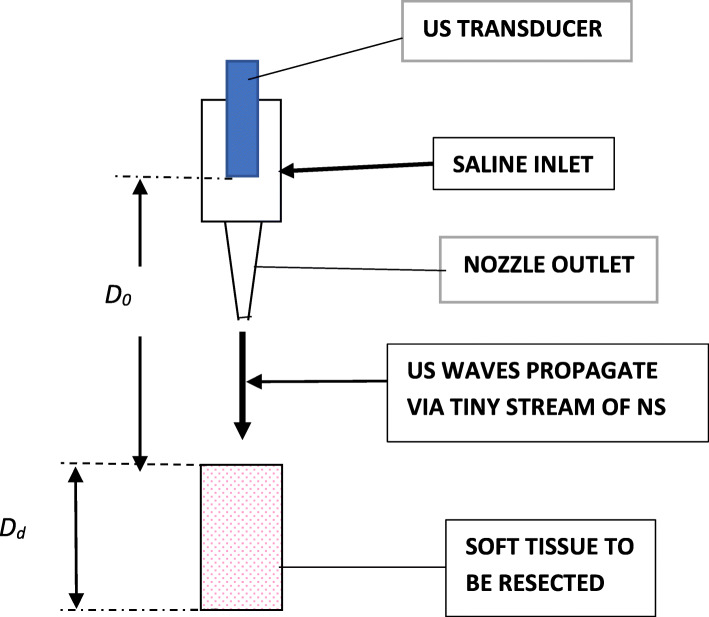
Schematic description of the nozzle in the “front end” (handpiece) of the novel US device

**Fig. 2:**
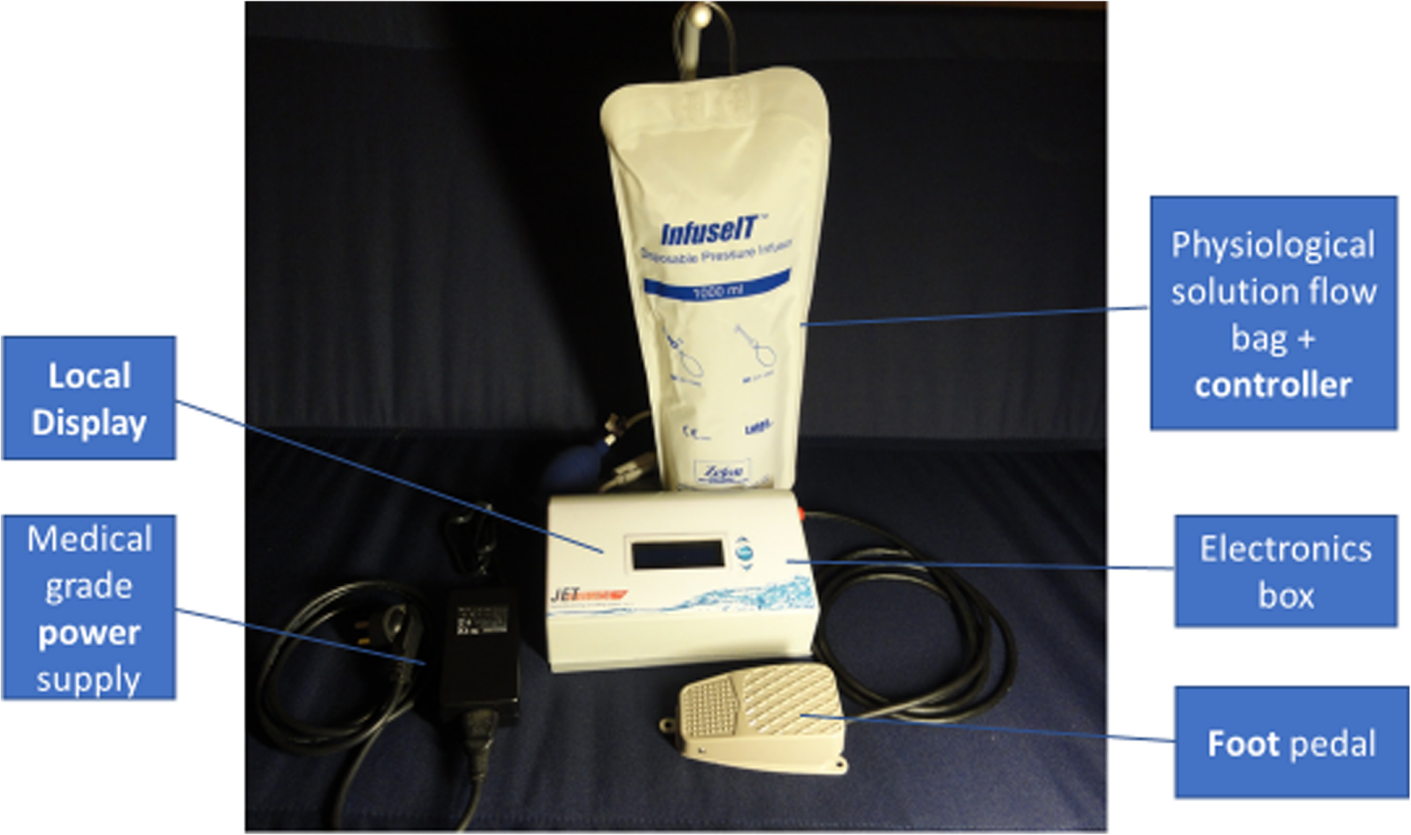
The novel US device (NUD)

In the presented study, we describe a new US device (NUD), as described in Figs. 1 and 2, that overcomes the mentioned drawbacks of the US imaging systems and its advantages during applications in neurosurgery; finally, a comparison of its data to those of a US imaging system is presented.

## Methods

The aim of this study was to present the NUD and to compare its properties and measuring data to those of a US imaging system.

### The new US measuring device

**Fig. 3:**
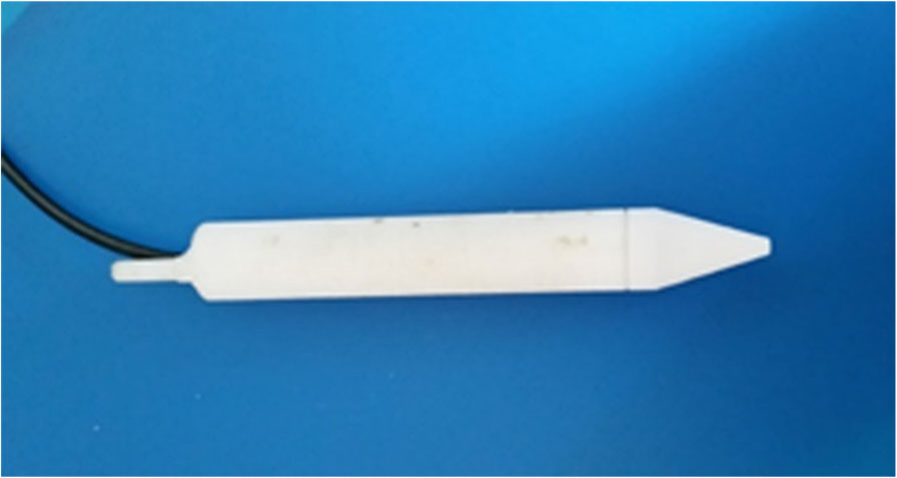
The hand held “front-end” (handpiece) of the NUD, for tumor-in-brain neurosurgeries

We describe here the NUD, including the monitoring methodology. The US waves (pulses) propagate from the handpiece of the NUD (Fig. 3) and through a tiny stream of NS (in a diameter of 1.3 mm) toward the investigated tissue. The information displayed graphically, describes the relative reflections of the US PE as a function of distance (depth), which is presented—together with the alpha-numeric one—on the same display. On the display are provided IO and simultaneously distances of tissue boundaries—information, which is crucial in neurosurgery. The NUD was applied successfully during tumor-in-brain neurosurgeries. With reference to Fig. 1, D_0_ is the distance between the active surface of the US transducer and the front surface of the soft tissue under investigation, and D_d_ is the distance from tissue’s front surface to its rear one (from where strong reflections are obtained) [11]. In Fig. 2, the pressurized sleeve with a bag of normal saline is located in the rear portion—behind the electronic unit. The air pressure in the sleeve was obtained by an air pump and controlled with a pressure gauge that provides a constant flow of saline from the nozzle. On the left side of the handpiece (Fig. 3) is the inlet of NS (through a sterilized pipe) and the location of the electrical cables (for the excitation and detection of US signals). On its right side (the conical part, i.e., the nozzle) is the outlet of NS that carries the US waves toward the investigated tissue. The NUD operates IO, in RT, was designed with a small footprint, and its data is obtained without any human intervention—therefore, it is obtained *objectively* and *quickly—*within less than 1 s. Usually, the stream of NS should be perpendicular to the tissue and about 4 mm above it (remote monitoring). However, angular inclinations of φ = ±7^0^ are acceptable (as data variations are according to cos^2^(φ)).

These neurosurgical clinical trials were performed after the research proposal was approved by the Helsinki committee (0307-09-RMB), the Israel Ministry of Health (HT-5534). Finally, the NUD received the regulatory approval, after it was tested successfully in an “Independent Laboratory” for safety, electro-magnetic compatibility (EMC), sterilization, biocompatibility, and against the intensity of the US pressure. Every neurosurgery that belonged to this research was performed only after explaining to the patient the additional measurements that were taken during the surgery and only after obtaining patient’s written and signed agreement in the “patient’s consent.”

The main feature of the NUD is its remote method of measurement; here, the US waves reach the “target” (tissue under investigation), and part of their echoes returns along the same path toward the US transducer. This feature enables one to monitor and control narrower regions and to direct the stream in a wider angular direction than allowed by other methods.

Assuming that the velocity of US is uniform within the same soft tissue, the distance of the echo is proportional to the time for the US waves to travel from the transducer to the interface and back again.

Lateral US resolution depends on the beam-width, and in the NUD, it is the width of the NS’s stream, i.e., 1.3 mm. The NUD overcomes (turns up-side down) the drawback of the traditional “compound scanning” (where the part to be examined is immersed in a liquid), since in this new device (with the tiny stream of NS that carries the US waves), it monitors at the small area that the stream of NS hits. Moreover, this stream has the ability to be directed in large angular directions.

The NUD consists of three main sub-units: *a handpiece* (Fig. 3), *electronics* (built mainly and around the data acquisition board, based on the EP2C5Q208C7 field-programmable gate array (FPGA) from the Altera Corporation, Cyclone®II family), and a *display* which is integrated in the “electronic unit” together with a connector in its rear part—for connecting a large display of the OR, via a designated cable. In the *electronic unit*, these signals are amplified, filtered, processed (digitally), and followed by algorithms that provide in real-time the information on the depth and the thickness of a tumor. These data are *displayed* simultaneously in graphical and alpha-numeric method; the NUD contains also an *audio* “approaching” signal, where a higher pitch represents shorter distance, which provides the neurosurgeon an additional information of approaching a tissue border.

#### The *methodology* of the performed experiments

In the first two experiments, we compared the measured data of the NUD with those of an US imaging system (GE VIVIDI, or BK Medical or Sonowand).

### Experiments—a chronologic description

**Fig. 4:**
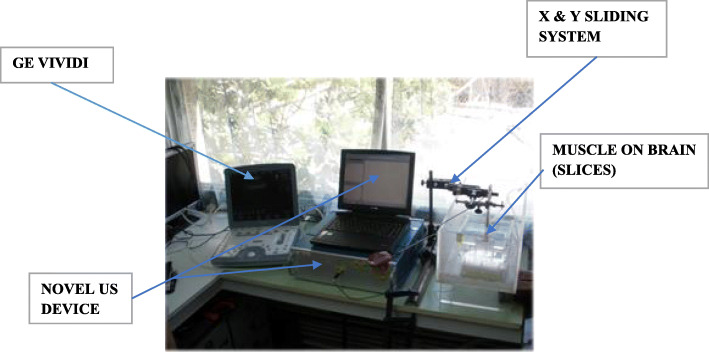
The measuring set-up of the laboratory ex vivo experiment

(i) A laboratory ex vivo trial (Fig. 4), where layered samples thicknesses (brain on muscle and vice versa, from a young porcine) were measured and compared with the NUD and an US imaging system (GE VIVIDI, or BK Medical, or Sonowand). The measuring set-up of this ex vivo laboratory experiment is described in Fig. 4. On the left side of Fig. 4 is placed the US imaging system (GE, type VIVIDI, where in other lab experiments, BK Medical, or Sonowand were applied); a prototype of the NUD is presented in the middle of this figure and on its right side—the mechanical system that enables the movement (sliding) of the handpiece in the x, y, and z directions.
(ii) Clinical human neurosurgical trials are presented, where we compared tumor depths, as measured with the NUD and an US imagining instrument (BK Medical or Sonowand), which are an integrated part of the operating room (OR);
(iii) Assessing tumor’s residual thickness during its resection. These measurements were performed solely with the NUD, after we obtained good correlations in stages (i) and (ii). Moreover, it was not feasible to stop resection, in order to pore NS in a restricted area, in order to obtain a good US conductivity and applying an US imaging transducer.

### Measuring details

#### General

We describe here the “round-trip” process of US propagation [11], initiated at the US transducer, its propagation to the “target” (tissue under investigation) and back to the same US transducer, where US waves are detected and transformed to electrical signals, followed by amplification, processing, and these signals are displayed.

The electronic pulser (located in the “electronics unit”) feeds the US transducer, causing its active surface (AS) to vibrate in an US frequency thus producing US waves in the NS. These US waves propagate in a “free jet” of a tiny laminar flow [11–17] and are incident on the investigated tissue and from there they are partially reflected and partially transmitted. The US pulse echoes (PE) are obtained from the front surface of that tissue and also from its rear boundary. These US echoes consist of specular and scattered reflections. The nature of specular reflections is that they propagate in a narrow angle. However, the scattered one is spread over a wide angle (due to the surface roughness relative to the US wavelength and the local reflection coefficient). Therefore, the relative intensity of the specular reflection is higher than the scattered one. Part of the US PE propagate back through the same tiny stream of NS and finally impinge the FS of the US transducer, where they are transformed to electrical signals [5, 9]. This process is followed by amplification, processing, and by a display (graphical and numerical).

A laboratory ex vivo experiment was *the first* set of experiments in this study. Here, we measured and compared thicknesses of fresh slices from the brain that was laid on a muscle or vice versa—both from a young porcine purchased from a slaughterhouse. The measurements were performed with the US imaging system (type VIVIDI, product of General Electric, USA), and in other experiments we applied BK Medical or Sonowand and the prototype of the NUD (Fig. 4). The display of the NUD presents in RT graphs of PE (expressed in volts), as a function of depths and provides simultaneously local thicknesses of the mentioned slices. This information is also displayed alpha-numerically.

*Note*: Comparison of depth and residual thickness measurements between those obtained with the NUD, a mechanical caliper (the Golden Standard) and those obtained from an X-ray (or a CT) image was presented [12–14, 16]. It was found there that the NUD and the mechanical measurements were very close, with differences not larger than 0.2 mm, with the same average and a smaller SD.

Taking these arguments into consideration, an ex vivo lab experiment was performed and is presented in the first part of this research. Depth and thickness assessed with the NUD and three types of US imaging instruments (GE VIVIDI, BK Medical, and Sonowand) are presented and summarized in Table 1.

**Table 1:** Summary of the average thicknesses and their differences (Δ), as obtained from measurements with an US imaging system (like GE, type VIVIDI) and the NUD

Experiment no. and type of US imaging system	Experiment type	US IMAGING SYSTEM		NUD		Difference Δ	
		Av. thickness				[mm]	
		[mm]					
		Muscle	Brain	Muscle	Brain	Muscle	Brain
**1 - GE VIVIDI**	**Muscle on the brain**	**6.3**	**8.2**	**7.7**	**7.2**	**1.4**	**1**
**2 - BK Medical**	**Muscle on the brain**	**8.7**	**7.1**	**7.6**	**7.2**	**1.1**	**0.1**
**3 - Sonowand**	**Muscle on the brain**	**10.4**	**6.5**	**9.6**	**5.8**	**0.8**	**0.7**
**4 -BK Medical**	**Brain on the muscle**	**8.2**	**8.5**	**8.1**	**8.3**	**0.1**	**0.2**
**5 - GE VIVIDI**	**Brain on the muscle**	**12.8**	**12.2**	**13.8**	**10.2**	**1**	**2**

In summary, for estimating the border between tissues, the NUD was found a more accurate device than an US imaging one.

##### Notes:

- During the preliminary stage of this study, thicknesses and depths were monitored and compared between the NUD and a mechanical caliper. For this purpose, a small plate of Perspex was inserted in a trabecular bone (TB). It was found that the difference between these measurements was not larger than ± 0.2 mm.
- The NUD has a fast (RT) response, where all the mentioned data is simultaneously displayed within less than 1 s, from the moment of initiating the measurement.
- While validating the NUD, its properties provide confidence to a neurosurgeon, and therefore it could potentially reduce malpractice complications while also truncating measuring time.

### Clinical trials

Human neurosurgical trials were performed during *the second* stage of this study. They were initiated after obtaining the approvals of the Helsinki committee of Rambam Health Care Center (HCC) and the Israel Ministry of Health and also the Regulatory one. Here, tumor’s depth was sequentially measured with the NUD and an US imaging instrument of the OR [SonoWand (SonoWand, Mison, Trondheim, Norway) or BK Medical (Bruel & Kjaer, Medical Division, Denmark)]. The presented data was obtained from 17 patients having a neurosurgery of a tumor-in-brain (type LGG). These patients were divided into 11 men and 6 women—all of them in the range of ages from 54 to 76 years. The correlation, associated between the two US measuring methods, was 87%, and the Pearson’s coefficient was 0.44 (*p* ≤ 0.05). This statistical analysis was obtained with the mentioned US imaging systems.

The *third stage* of this study was initiated after we analyzed the results of the second stage and taking into consideration the good correlation obtained between the measured data by these two US monitoring devices. In the third stage, tumor thickness was monitored and controlled IO during resection. Due to its critical nature, the surgery was performed by the most senior staff of the Neurosurgery Department. Due to the unique properties of the NUD that were mentioned earlier, it was the only one applied in this stage of neurosurgery.

According to the preliminary experiments mentioned above and those of stages I and II of this study, the NUD was found a reliable and accurate device for assessing IO and in RT the border between tissues. Its ability to monitor remotely, together with its small footprint, assisted during this delicate part of the surgery—thus obtaining better decisions of resection boundaries.

### Experiments

Described here are the experiments that were performed during this study.

#### Ex vivo experiment

A fresh slice of muscle was laid on a fresh slice of a brain—both from a young porcine. Their individual and total thicknesses were assessed using an US imaging system (GE VIVIDI, or BK Medical, or Sonowand) and the NUD. While measuring with one of the US imaging systems, the slices were immersed in NS, in order to obtain a good US conduction; while with the NUD—the investigated small region on the slice was wetted by the tiny stream of NS (which is an integrated part of the NUD), through which the US waves propagate (Figs. 1, 2, and 3). Accordingly, the required US conduction was achieved automatically and without interfering the surgery.

**Fig. 5:**
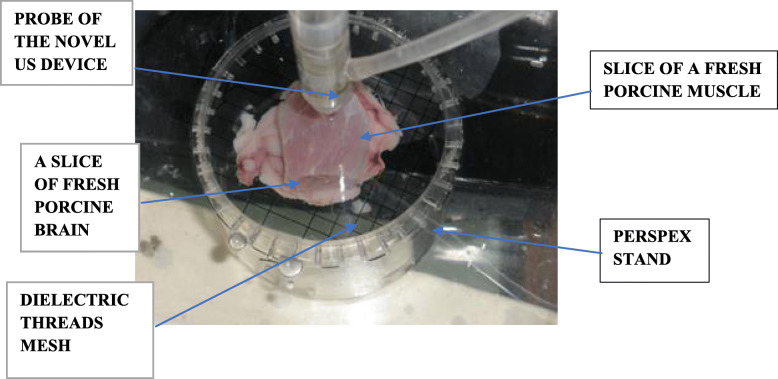
Porcine slices laid on mesh, with nozzle above them

The slice of brain (or muscle) was laid on a mesh (made of a thin dielectric wire of thickness 0.5 mm, where the size of an elementary square was 5 × 5 mm), which was clamped to a circular stand, mage of Perspex (Fig. 5). Above the slices, at a height of 4 mm, is shown, in that figure, the nozzle of the handpiece (prototype), which is a part of the NUD (thus, it measures *remotely*). The inlet for NS is on the side of the probe (made of a dielectric pipe). The NS flows out the nozzle in a tiny stream, toward a slice. The US waves (pulses) propagate through this tiny stream of NS toward the slice, while the reflected part in the opposite direction.

Five experimental groups were performed. In three of them, a fresh slice of a porcine muscle was laid on top of a fresh slice of a porcine brain and in the other two cases—the opposite layering was applied.

**Fig. 6:**
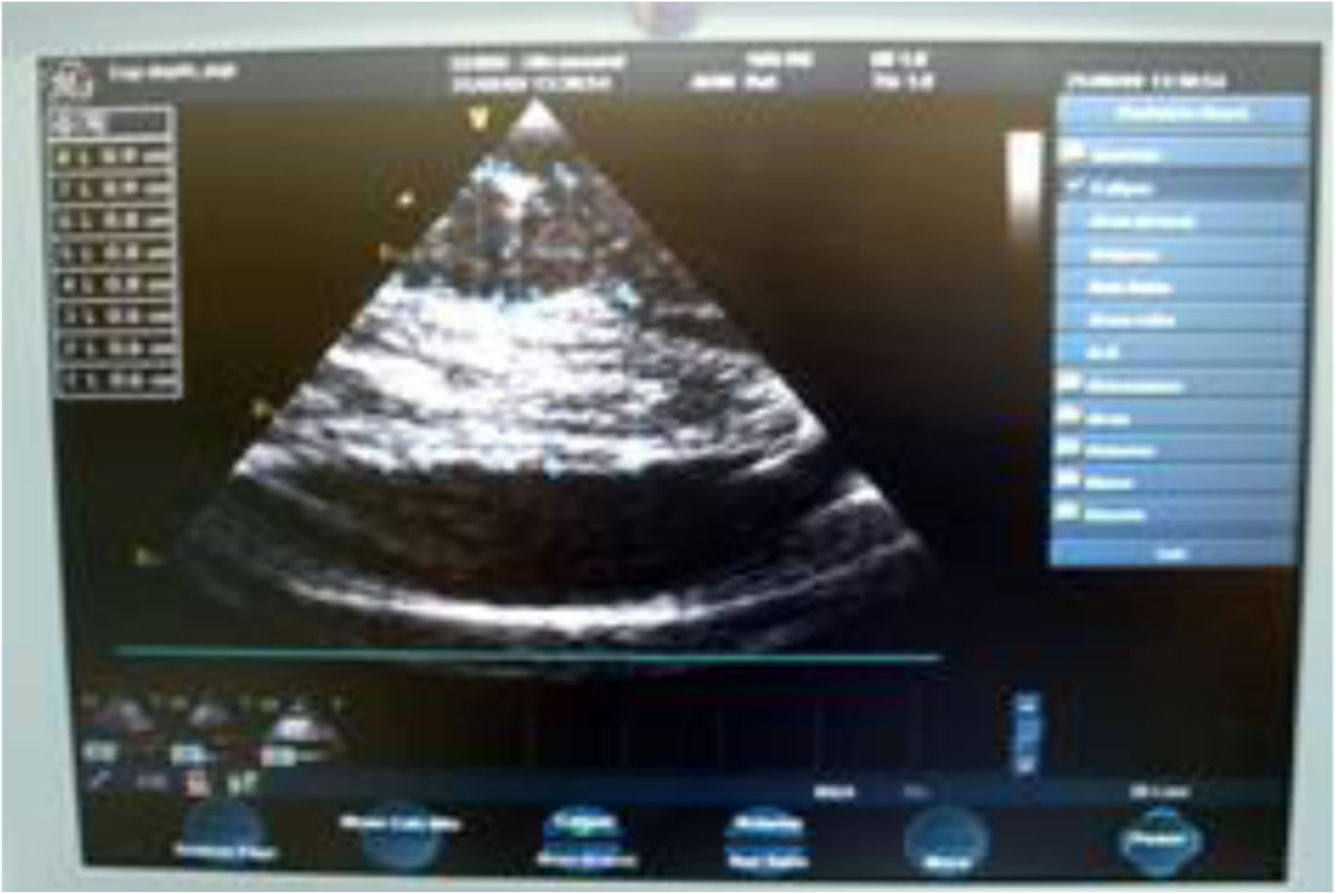
An US image describing fresh slices of muscle on brain, from a young porcine

The thicknesses of these slices were also measured with one of the US imaging systems (GE VIVIDI, BK Medical, or Sonowand). Figure 6 is an example of the obtained US image that was obtained with GE VIVIDI, where a slice of fresh muscle was laid on a slice of fresh brain. The slice of the fresh brain was laid on a thin dielectric mesh, partially inserted in NS. The individual and total slice thicknesses were measured manually from the US image, using the built-in electronic ruler. The NUD provided simultaneously and in RT the upper, lower, and total slice thicknesses.

Thicknesses slice variations were measured with the NUD, in steps of 3 ± 0.1 mm along a virtual straight line above the upper slice and at a height of 4 ± 0.5 mm from the slice. For monitoring along the slice, a sliding mechanism was applied, as described on the right side of Fig. 4.

#### Assessing tumor depth during clinical human neurosurgeries:

The neurosurgeon obtains information about tumor’s location, size, and the type of soft tissues surrounding it, by analyzing the pre-operative images obtained through a computer tomographic imaging (CTI called also CT) and a magnetic resonance imaging (MRI). After the surgery was initiated and after patient’s skull bone was partially removed, shifts of the brain and tumor occur due to cerebrospinal fluid drainage, tissue removal, and the gravity. They introduce significant inaccuracies [10] that may render the surgery result. A more accurate and updated tumor position and location is required for a successful neurosurgery, which is provided at this point by applying an US monitoring device.

In the presented clinical trials, an US imaging instrument of the OR (Sonowand or BK Medical) was used, together with the NUD. It enabled the neurosurgeon to compare their data as it was used under the same operating conditions. When the US imaging system was applied, tumor’s depth was measured on the instrument’s display. This kind of measurement is not performed by the senior neurosurgeon but rather by a dedicated staff member of the OR, and it is therefore a *subjective* method. Moreover, this data was not obtained in RT, and the measuring accuracy was not better than ± 1.5 mm. At the same place and conditions, the NUD provided tumor’s depth instantly and directly to the neurosurgeon with an accuracy better than ± 0.3 mm, and its results are of an *objective* nature—as there are no human interventions during its data processing.

According to the measurements performed with the NUD and were compared to the US imaging system, the NUD enabled a fast (≤ 1 s) and less ambiguities of the assessed data.

#### Assessing tumor thickness (during its resection)

The remote monitoring capability and the small footprint of the NUD enabled its application during tumor’s resection. Due to the glioma’s nature, this part of a neurosurgery is frequently performed in narrow and confined regions. Thus, it is performed by senior neurosurgeons, where the NUD provides them directly (IO and in RT) the *residual* tumor thickness. Figure 3 describes the neurosurgical hand held unit (handpiece); from the end of the conical part (the nozzle) is the stream outlet of NS, through which the US waves propagate.

## Results

### Laboratory ex vivo experiment

Table 1 summarizes the average slice thicknesses and their differences, by comparing the measuring data obtained with an US imaging system and the NUD. When measuring with the NUD, the maximum SD was ± 0.18 mm and with the US imaging system ± 0.8 mm.

### Remarks to Table 1

#### Experiment 1

Thicknesses comparison of the muscle and brain slice was performed by measuring them with the US imaging system (GE, type VIVIDI) and the NUD. The average difference in slice thickness of the muscle was 1.4 mm and for slice of the brain was 1 mm.

#### Experiment 2

Similar difference was obtained in slice thickness measurement for the muscle, while for the slice of the brain the difference in thickness was not larger than 0.1 mm. The measurements were performed with BK Medical US imaging system and the NUD.

#### Experiment 3

Maximum difference of 0.8 mm was obtained for the average thickness of the muscle and 0.7 mm for slice thickness of the brain. The measurements were performed with Sonowand US imaging system and the NUD.

#### Experiment 4

Maximum difference in slice thickness of 0.1 mm was obtained for a muscle and 0.2 mm for a slice of the brain. The measurements were performed with BK Medical US imaging system and the NUD.

#### Experiment 5

Maximum difference in slice thickness of 2 mm was obtained for the muscle and 1 mm for a slice of the brain. The measurements were performed with GE VIVIDI US imaging system and the NUD.

### Clinical human trials

The clinical human trials were divided in two parts, according to the subject’s stage in neurosurgery as follows:

(i) During the *first stage* of neurosurgery, data of tumor’s depth was compared with those of the US imaging systems of the OR and the NUD.
(ii) During the *second stage* of the neurosurgery, when tumor (glioma) resection took a part, its residual thickness was monitored solely with NUD.

*Tumor depth* was obtained during the first stage of neurosurgery, when the US imaging systems of the OR and the NUD were applied.

**Fig. 7:**
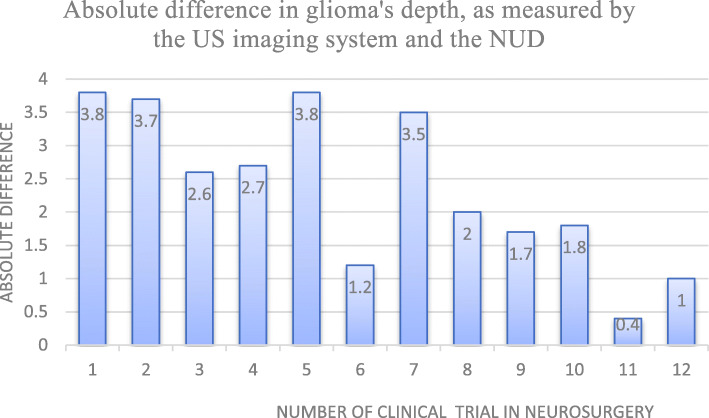
Absolute difference of glioma’s depth, as measured by the US imaging system and the NUD

Figure 7 describes the absolute differences of glioma’s depths as measured with the US imaging system (Sonowand, or BK Medical) of the OR and the NUD. The abscissa describes the number of compared measurements, and the ordinate—the absolute difference between their values.

Table 2 presents the means, ranges, and standard deviations (SD) for the mentioned measuring methods and the differences between them. The table presents means, ranges, and standard deviations (SD) for these US devices and the differences between them.

**Table 2:** Glioma’s depth (from dura), as measured with the US imaging system (Sonowand, or BK Medical) of the OR and the NUD

	A = US imaging	B = NUD	C = |A − B|
**Mean (mm)**	**18.35**	**18.8**	**0.45**
**SD (mm)**	**1.8**	**0.6**	**1.2**
**Depth rage (mm)**	**16.55; 20.15**	**18.2; 19.4**	**16.5; 0.75**

It was found from Table 2 that the mentioned US measuring methods provide similar (97%) glioma’s depths, supported also by the Pearson’s correlation coefficient 0.44 (*p* ≤ 0.03), associated between these measuring techniques.

Table 3 presents several measuring results that were obtained during a glioma resection. Each measurement was repeated 5 times, and their average and SD values are presented.

**Table 3:** Residual thickness during glioma’s resection, as monitored with the NUD, at four stages of resection

Resection case no.	Measuring case no.	Residual tumor thickness mm	SD of tumor thickness mm
**1**	**1**	**18.6**	**± 0.7**
	**2**	**17.2**	**± 0.5**
	**3**	**5.6**	**± 0.4**
	**4**	**3.4**	**± 0.3**
**2**	**1**	**12**	**± 0.5**
	**2**	**6.7**	**± 0.4**
	**3**	**5.5**	**± 0.4**
	**4**	**3.8**	**± 0.3**
**3**	**1**	**15.7**	**± 0.6**
	**2**	**12.3**	**± 0.5**
	**3**	**7.2**	**± 0.4**
	**4**	**3.4**	**± 0.3**
**4**	**1**	**17.8**	**± 0.7**
	**2**	**11.4**	**± 0.6**
	**3**	**6.5**	**± 0.5**
	**4**	**3.2**	**± 0.4**

Table 4 presents several cases of residual tumor thickness, before final resection was performed, as monitored with the NUD. After the final resection, no residual glioma’s thickness was monitored nor viewed by the neurosurgeon.

**Table 4:** Four cases of the average residual glioma’s thickness in the final stage of resection, as measured with the NUD

Residual resection case	Residual resection stage	Av. residual glioma thickness [mm]	SD of residual glioma thickness [mm]
**1**	**1**	**2.7**	**± 0.3**
	**2**	**2.2**	**± 0.25**
	**3**	**1.7**	**± 0.2**
**2**	**1**	**2.6**	**± 0.3**
	**2**	**2.3**	**± 0.25**
	**3**	**1.8**	**± 0.2**
**3**	**1**	**2.4**	**± 0.25**
	**2**	**2.1**	**± 0.25**
	**3**	**1.9**	**± 0.2**
**4**	**1**	**2.3**	**± 0.3**
	**2**	**2**	**± 0.25**
	**3**	**1.8**	**± 0.2**
	**4**	**1.6**	**± 0.2**

### Graphical presentation of glioma’s residual thickness during its resection

In this section are presented graphs of glioma residual thickness, for several stages of glioma resection, as monitored with the NUD.

#### Graphs nomenclature

In these graphical presentations, the ordinate (*y*-axis) represents relative reflected signals (Volts), and the abscissa (*x*-axis) represents distance/depth, which are measured in “samples” (1 sample = 14.3·10^−3^ mm). AB represents glioma thickness; *M* is the signal obtained at nozzle’s outlet, where the NS flow passes from a “bounded” to a “free space” regions.

**Fig. 8:**
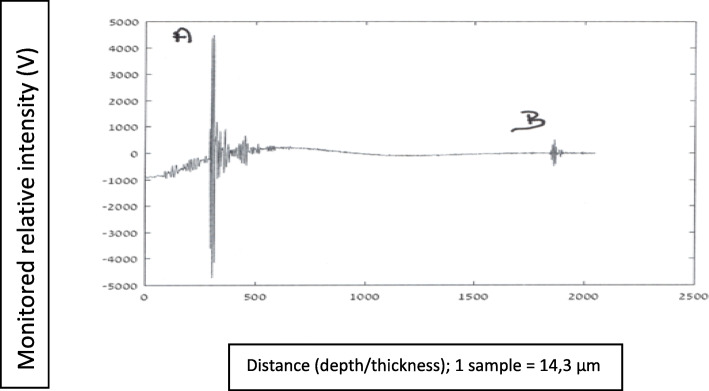
Glioma’s thickness (Δ×AB = 22.2 mm), measured with the NUD

**Fig. 9:**
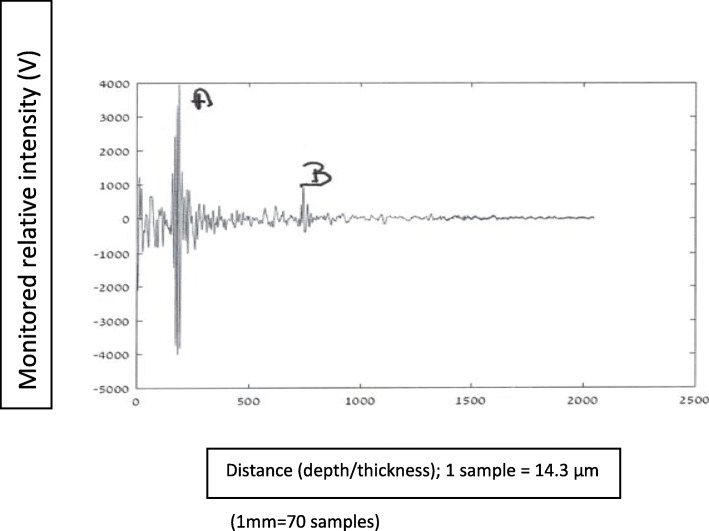
Glioma thickness (Δ×AB = 8 mm) during its resection, measured with the NUD

**Fig. 10:**
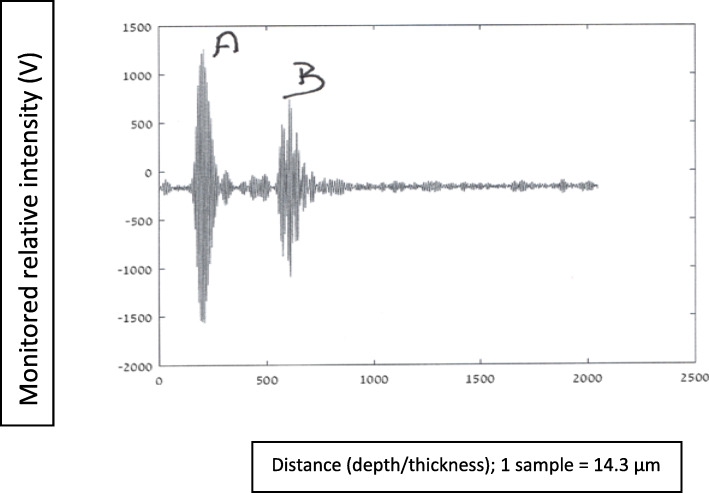
Glioma thickness (Δ×AB = 5.2 mm) during its resection, measured with the NUD

**Fig. 11:**
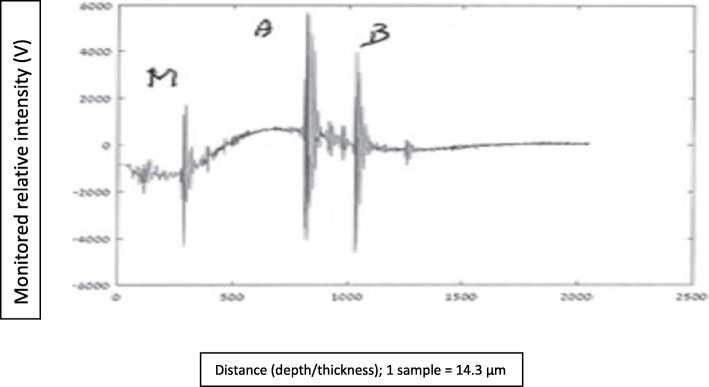
Glioma thickness (Δ×AB = 3.7 mm) during its resection, measured with the NUD

**Fig. 12:**
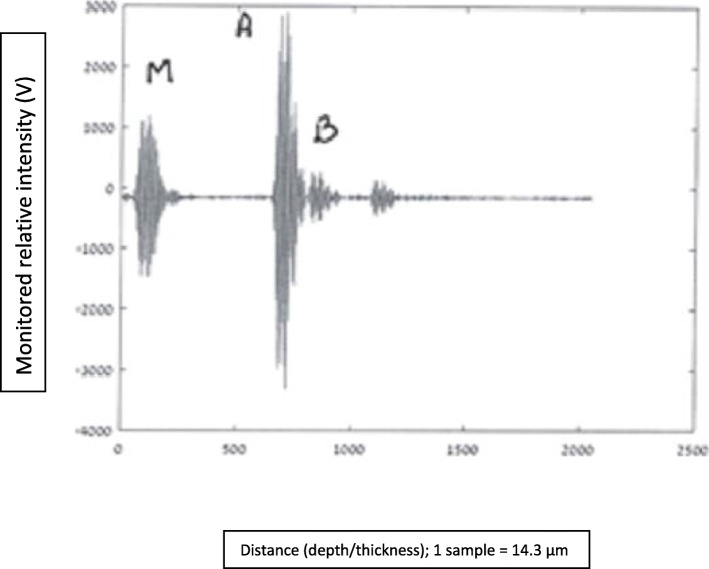
Glioma thickness (Δ×AB = 2 mm) during its resection, measured with the NUD

Figures 8, 9, 10, 11, and 12 describe glioma residual thickness during its resection, from Δx_AB_ = 22.2 to 2 mm, respectively.

## Discussion

The NUD was successfully applied to obtain IO and in RT the distance of tissue boundaries during monitoring a tumor (glioma) in the brain.

The ability of this new device to monitor in vivo and IO proved successfully its ability to evaluate glioma’s depth with a better accuracy than the US imaging system. It was also proved that the NUD monitors IO glioma’s residual thickness during its resection. As the NUD monitors in highly restricted areas of the brain, it provides a new ability in neurosurgery.

Advantages of the NUD were clearly demonstrated in the ex vivo experiments and also during all the human clinical trials. In these clinical trials, the NUD was applied only for a glioma; it is therefore suggested that researchers continue this investigation on other types of tumors as well, especially since a glioma is an invasively growing tumor that is exceptionally difficult to separate from healthy brain tissue. We also recommend further investigation on different population sample sizes and of different ages.

### Specific remarks

#### Glioma’s depth

Glioma’s depth was measured with the NUD and compared to the US imaging system of the OR (Sonowand or BK Medical). The difference between their absolute values was calculated, according to (Δ|US_image_ − US_NUD_**|**), as described in Fig. 7. Differences between 0 and 2.5 mm are within 6.7%, and 33% are between 2.5 and 4 mm. The correlation coefficient between these measurements was 87%, meaning that they were also within that span and the Pearson’s correlation coefficient (0.44) and supports this consequence and values of the *mean* and its *SD* that were similar for these measurements.

#### Glioma’s residual thickness

The ability of the NUD to monitor IO and in RT glioma’s residual thickness is required mainly in the third stage of neurosurgery. This ability was achieved due to its specific features: (i) small footprint of its handpiece (Fig. 3) and the wide angular directivity of its stream and (ii) the fast response of the NUD that provides in RT the processed data. These properties of the NUD enabled to monitor glioma’s residual thickness IO and in RT. Consequently, they assisted to prevent unnecessary resections of the healthy brain tissue. This was found especially important during the stage of tumor resection, as the NUD has a great benefit of being a “safety tool” that enables a surgery outcome to become with better results.

### Features of the NUD and the imaging system

- To define glioma’s depth and thickness with an US imaging system, it is necessary to interrupt the surgery procedure, in order to pore the NS. Moreover, the assessment is performed subjectively by measuring distances (lengths/depths) on instrument’s display that presents an US image. Thus, it is not a RT method or an objective one, and it slows down the surgical process [10].
- In many clinical cases, where monitoring resection in restricted and narrow spaces is essential, transducer’s diameter (in US imaging systems) was found in many clinical cases too large [10].
- The NUD has the ability to monitor successfully the resection, as demonstrated during all stages of neurosurgery, and it was used until tumor’s complete removal was achieved. In this final stage, the control was also done by surgeon’s visualization.

## Conclusions

The ability of the NUD to monitor IO glioma’s depth, with a better accuracy than an US imaging system, enables its application in neurosurgery. Furthermore, it also provides its data in RT and without an operator dependence. The average thickness of slices and their SD in the ex vivo experiment were closer between the NUD and BK Medical than to Sonowand.

The advantages of the NUD, by monitoring IO in RT and providing all the data simultaneously and objectively, were clearly demonstrated in the trials of these studies.

It was also proved that the NUD monitors IO and in RT glioma’s residual thickness during its resection.

As the NUD is able to monitor glioma’s thickness in restricted areas, it provides a new method in carrying out neurosurgery. Thus, it can improve the surgical outcomes, as it enables the reduction of human error in these delicate cases.

## Data Availability

Not published data and materials will be available under request.

## Abbreviations

AS: Active surface
AT: Attenuation
CT: Computer tomography
CTI: Computer tomographic imaging
FPGA: Field programmable gate array
IO: Intraoperative
FS: Front surface
LGG: Low-grade glioma
MRI: Magnetic resonance imaging
NS: Normal saline
NUD: New ultrasonic device
OR: Operating room
PE: Pulse echo
RT: Real-time
Re: Reflection
SOS: Speed of sound
Tr: Transmission
US: Ultrasound
